# Prevalence of hepatitis B viruses and associated factors among pregnant women attending antenatal clinics in public hospitals of Wolaita Zone, South Ethiopia

**DOI:** 10.1371/journal.pone.0232653

**Published:** 2020-05-07

**Authors:** Belete Bancha, Aseb Arba Kinfe, Kebreab Paulos Chanko, Shimelash Bitew Workie, Takele Tadese

**Affiliations:** 1 School of Medical Laboratory Science, College of Medicine and Health Sciences, Wolaita Sodo University, Wolaita Sodo, Ethiopia; 2 School of Nursing, College of Medicine and Health Sciences, Wolaita Sodo University, Wolaita Sodo, Ethiopia; 3 School of Midwifery, College of Medicine and Health Sciences, Wolaita Sodo University, Wolaita Sodo, Ethiopia; 4 School of Public Health, College of Medicine and Health Sciences, Wolaita Sodo University, Wolaita Sodo, Ethiopia; university of campus biomedico, ITALY

## Abstract

**Background:**

Hepatitis B virus (HBV) infection is a serious public health problem in sub-Saharan Africa pregnant women. HBV Infection has high tendency of vertical transmission and have adverse effect on both the mother and child. However, there is no evidence on prevalence of hepatitis B virus among pregnant women in Wolaita Zone. Therefore, this study aims to determine prevalence and associated factors of hepatitis B virus infection among pregnant attending Antenatal clinics of public Hospitals of Wolaita Zone.

**Method:**

An institution based cross sectional study was conducted among pregnant women attending antenatal clinics of Wolaita Zone from October—November, 2018. Systematic random sampling was used to get respondents. A pretested, structured questionnaire was used to collect socio-demographic characteristics and other variables. In addition, 5 ml of venous blood was collected from each study participants and serum was tested for Hepatitis B surface antigen. Data was entered to Epidata 3.1 version and exported Statistical Package for Social Sciences Version 20.0 for descriptive and logistic regression analysis. All variables in bivariate analysis with p<0.25 were taken to multivariable analysis. P-value and Odds ratio with 95% CI was used to measure the presence and strength of the association respectively.

**Result:**

The prevalence of Hepatitis B surface Antigen among pregnant women was 49(7.3%). History of multiple sexual partners (AOR = 2.675, 95%CI = 1.107–6.463), surgical procedure (AOR = 3.218, 95%CI = 1.446–7.163), genital mutilation (AOR = 2.72, 95% CI = 1.407–5.263), and tooth extraction (AOR = 2.049, 95%CI = 1.061–3.956) were statistically associated with HBV.

**Conclusion and recommendation:**

Intermediate endemicity of Hepatitis B Virus (7.3%) was observed among mothers attending antenatal clinics of Wolaita Zone. History of tooth extraction, history of surgical procedure, history of genital mutilation and history multiple sexual partners were factors associated with acquisition of Hepatitis B Virus infection. Therefore, we recommend that the health education programs should be done to avoid traditional and non-sterile tooth extraction methods, female genital mutilation and avoiding having multiple sexual partner and its consequences to community and to raise the awareness of mothers attending antenatal clinics. Facilities should strictly follow sterile procedures in every surgical procedure.

## Introduction

Hepatitis B infection is a potentially life-threatening liver disease caused by hepatitis B virus. Viral hepatitis is an inflammation of the liver by viruses affecting millions of people every year. Among five different types of hepatitis viruses, the most common virus that affects liver is Hepatitis B viruses [1].

It is a major global health problem and the most serious type of viral hepatitis. It can cause chronic liver disease and put people at high risk of death from cirrhosis of liver and liver cancer [2]. Infection with hepatitis B virus (HBV) poses a public health burden, as it is 50–100 times more contagious than human immune deficiency virus (HIV) [3]. Hepatitis B Virus (HBV) is a main cause of morbidity and mortality, touching almost every class of person and age group with vertical transmission being the commonest route of transmission in many endemic areas [4].

Viral hepatitis during pregnancy is associated with high risk of maternal complications and high rate of vertical transmission. Fetal and neonatal hepatitis acquired from mother during pregnancy lead to impaired cognitive and physical development in later life of the children. Neonatal contamination does occur during labor and delivery [5]. Vertical transmission from chronic carrier mothers exceeds 90% and accounts for up to 40% of the world carriers in endemic areas. If contaminated, the neonate becomes a chronic carrier himself in 80% to 90% of cases and is prone to cirrhosis and hepatocellular carcinoma in adult life [6]. The risk of prenatal HBV transmission is greatest for infant born to women who are Hepatitis B e antigen positive with infective rate of 70% to 90% at age of 6 month and about 90% children remains chronically infected without intervention [7].

The factors for hepatitis infection are known to be linked to body fluids especially those with high concentration of the virus like blood, semen and vaginal secretions. Traditional practices that expose people to hepatitis B infection like scarification, ear and nose piercing as well as tattoos have led to higher prevalence in certain zones but not only in pregnancy [8].

Hepatitis B virus (HBV) infection is a major global health problem, especially in Asia, Africa, southern Europe and Latin America. Over 20 million people are infected annually with this virus globally and there are 350–400 million chronic carrier of Hepatitis B virus (HBV)[9]. Globally, there are 400 million people infected with HBV, and the risk continues to rise as prenatal and early childhood infections revamp which risks over 95% of the infected persons to change to chronicity [10].

Even though, it is difficult to identify the exact burden of HBV in Africa, between 56% and 98% of the adult population show evidence of past exposure to HBV infection and the sero-prevalence of hepatitis B surface antigen (HBsAg) has been estimated to range from 6% to 20%. According to WHO 2017 report, HBV infection in pregnancy can result in occurrence of pre-term delivery and low birth weight in addition to vertical transmission. Furthermore, HBV infection has been reported to be associated with threatened preterm labor, antepartum hemorrhage as well as gestational diabetes mellitus [11, 12, 13].

However, there is no evidence on prevalence of hepatitis B virus and associated factors among pregnant women in Wolaita zone. Therefore, the objective of this study was to determine the prevalence of the sero-prevalence of Hepatitis B and to identify factors associated with hepatitis B virus infection among pregnant mothers attending antenatal clinics (ANC) in Wolaita Zone at public Hospitals.

## Methods and materials

### Study setting

The study was conducted in the public hospitals of Wolayta zone of Southern Ethiopia. Administratively, Wolaita Zone is divided in to 12 districts and 3 city administrations with 336 kebeles which is lowest administrative structure in Ethiopia.

The total estimated number of pregnant women in the zone was 68306. There were 5 public hospitals in the Wolaita Zone. The study was conducted on three public hospitals in the Zone.

### Study design and period

An institution based cross sectional study was conducted among pregnant women attending ANC clinic at selected public hospitals of Wolayta Zone from October-November, 2018.

### Population

All pregnant women attending ANC clinics in the public hospitals of Wolaita Zone were considered as source population, whereas all pregnant women attending antenatal clinics of selected public hospitals of Wolayta zone were taken as study population and pregnant woman selected for study from each hospital as study unit.

### Inclusion and exclusion criteria

All pregnant women whose pregnancy is confirmed by clinical history and examination or an obstetric ultrasound scan were included in study. Pregnant women who are critically sick and unable answer the questionnaire during data collection were excluded from study.

### Sample size determination

Sample size was calculated for both objectives by using Open-EPI sample size calculator software using 7.8% prevalence of HBV [14], a 3% margin of error, a design effect of 2, and a non-response rate of 10%. The final calculated sample size was 675.

### Sampling technique

There are five public hospitals in Wolayta zone. Three public hospitals were randomly selected and got annual and quarter client flow data from each selected hospitals. Proportional allocation was done to each public hospitals taking monthly client flow of each hospitals. Systematic sampling technique was used to get samples.

### Study variables

#### Dependent variables

Sero-prevalence of HBV

#### Independent variables

Family history of HBV, history of abortion, history of multiple sexual partners, body tattooing, surgical procedure, hospital admission, genital mutilation, gestational age, sex, marital status, occupation, educational status, history of blood transfusion, place of pervious birth, residence, income, history of sharing sharp instruments, tooth extraction and alcohol consumption.

#### Data collection tools and procedure

A pre-tested structured and interviewer administered questionnaire was delivered to eligible pregnant woman was interviewed to obtain socio-demographic information including maternal age, gestation age, occupation, residence, highest level of education and other information on risk factors for transmission of HBV, including a history of previous blood transfusions, Genital mutilation, place of previous birth, any surgical procedure, tattooing, and hospital admission, sharing of sharp materials, tooth extraction, alcohol consumption, family history of HBV and history of multiple sexual partner by trained midwives and nurse.

The women coming after service in ANC clinic were asked to participate in the study by the data collectors. Following their consent, each woman was interviewed individually and blood sample was taken. A data collector was ensuring that all mothers coming out of ANC clinic are appropriately directed for study inclusion. The data collector then picked another woman after completing first interview.

#### Laboratory testing methods

5ml of venous blood was drawn under aseptic conditions in disposable vacuntainer tubes by experienced laboratory personnel. The rapid test was performed to deliver the result of the pregnant women at the time of screening. Sample testing for HBsAg was done using bioline strip test which has specificity and sensitivity of greater than 99%.

The membrane is pre-coated with anti-HBsAg antibodies on the test band region and anti-mouse antibodies on the control band region. During testing, the serum sample reacts with the dye conjugate (mouse anti HBsAg antibody colloidal gold conjugate) that will be coated in the test strip or caste. The mixture then by capillary action reacts with anti-HBsAg antibodies on the membrane and generates a red band. Presence of this red band indicates a positive result while its absence indicates a negative result (27).

#### Quality assurance

Questionnaire was translated into Amharic language then back to English. Pretest was done on 5% of the sample size at Sodo health center 1week before actual data collection. Necessary modifications were made on the questionnaire after conducting the pretest. Close supervision was done and standardized procedures were strictly followed during blood sample collection, storage and analytical process.

Positive and negative control samples within the test kit were run to assess the performance of the test kit as internal quality control. Known positive and negative serum samples for HBsAg confirmed by enzyme linked immunosorbent assay (ELISA) technique was obtained from Ethiopian Blood Bank, Sodo branch. This known serum sample was analyzed before the actual investigation as external quality control of the test kit. The completeness and consistency of questionnaires was checked every day by principal investigator and supervisor on each day of data collection.

### Operational definition

**HBsAg Positive-**Two distinct red bands appear, on in test region and another in the control region. **HBsAg Negative—**A single red band appears in the control region. No apparent red or pink band appears in the test region. **HBsAg Invalid—**Control band fails to appear which means improper testing procedure or deterioration of reagents probably so that the test should be repeated. **Multiple sexual partners:** A person who had engaged in sexual activity with two or more partner in a 12 month time period.

### Data management analysis procedure

After checking for the completeness, accuracy and clarity of the data collected, the data was entered into Epi Data version 3.1. Then, it was exported to statistical package for social science (SPSS) software version 20 for analysis. Summary statistics such as frequencies and percentages were computed. Bivariate analysis was conducted to differentiate the variables that will become candidate for multivariable analysis.

Variables with P-value less than 0.25 in bivariate analysis were entered into multivariate analysis to control for possible effect of confounder. The multi collinearity effect and Hosmer and Lemeshow checked for model fitness. The variables with p-value less than 0.05 in the multivariable logistic regression were declared to having significant association with outcome variable. Odds ratio with 95% CI was used to measure the strength of the association.

### Ethical consideration

The study was approved by the Institutional Review Board of Wolaita Sodo University. Then official ethical approval letter was written in reference number of CARD 32/31/2018 to respective hospitals. The study was conducted according to declaration of Helsinki. Interviews were conducted in private place after taking informed written consent. Test results were given to the clinicians who are working on ANC clinic of the Hospital for further diagnosis and management. All findings were kept confidential and remaining blood sample was not used for any other purpose.

### Search terms and strategy

Search words used were Hepatitis B virus, factors, hepatitis B virus surface antigen, and pregnant women using PUBMED, MEDLINE.

## Results

### Socio-demographic characteristics

A total of 675 women participated in the study making the response rate of 100%. The mean age was 26 years with standard deviation of ±4.22. 42.8% and 28.4% of respondents were in the age category of 23-27years and age category 28-32years. Four hundred forty four (65.8%) of the respondents were urban dwellers. The majority of the study participants were housewives that accounts 272 (40.3%), followed by employee 196 (29%) and merchants 124(18.1%).

Regarding level of education, 229(33.9%) of the women learned to the level of diploma and above whereas 126(18.7%) had no formal education. Concerning income, 275(40.7%) of participants had monthly income of 1501 ETB and above. Majority of participants were Wolayta 526(77.8%), followed by Gurage, which accounts 44% in ethnicity as shown in Table 1.

**Table 1:** Socio-demographic characteristics of pregnant women attending antenatal care clinics of selected public hospitals in Wolayta zone, 2018 (N = 675).

Variable(n = 675)	Category	Frequency	Percent (%)
**Age of respondents**	18–22	157	23.3
	23–27	289	42.8
	28–32	192	28.4
	> = 33	37	5.5
**Residence**	Urban	444	65.8
	Rural	231	34.2
**Marital status**	Single	55	8.1
	Ever married	620	91.9
**Occupation**	Employee	196	29
	Merchant	124	18.4
	Farmer	19	2.8
	Daily laborer	64	9.5
	Housewife	272	40.3
**Educational level**	No formal education	126	18.7
	Primary education	127	18.8
	Secondary education	193	28.6
	Diploma and above	229	33.9

### Route associated factors of HBV infection

From a total of 675 study participants, 72(10.7%) had history of surgical procedure performed on them, 57(8.4%) had history of blood transfusion, 178(26%) had history of tooth extraction and 492(72.9%) had history of genital mutilation. Forty nine (7.3%) responded presence of HBV in their family members. Among 675 participants, 48(7.1%) had history of multiple sexual partners, 170(25%) had history of abortion, 25(3.7%) respondents shared sharp material as shown in Table 2.

**Table 2:** Risky practices of HBV infection among pregnant women attending antenatal clinics of public hospitals in Wolayta zone, 2018.

Variables(n = 675)	Category	Frequency	Percent (%)
Surgical procedure	Yes	72	10.7
	No	603	89.3
History of blood transfusion	Yes	57	8.4
	No	618	91.6
Tooth extraction	Yes	178	26
	No	497	73.6
History of multiple sexual partner	Yes	48	7.1
	No	627	92.8
Genital mutilation	Yes	492	72.9
	No	183	27.1
Family history of HBV	Yes	49	7.3
	No	626	92.7
Body tattooing	Yes	83	12.3
	No	592	87.7
History of abortion	Yes	170	25
	No	505	74.8
Sharing sharp material	Yes	25	3.7
	No	650	96.3
Hospital Admission	Yes	197	29.2
	No	478	70.8

### Prevalence of HBV infection

**Fig 1:**
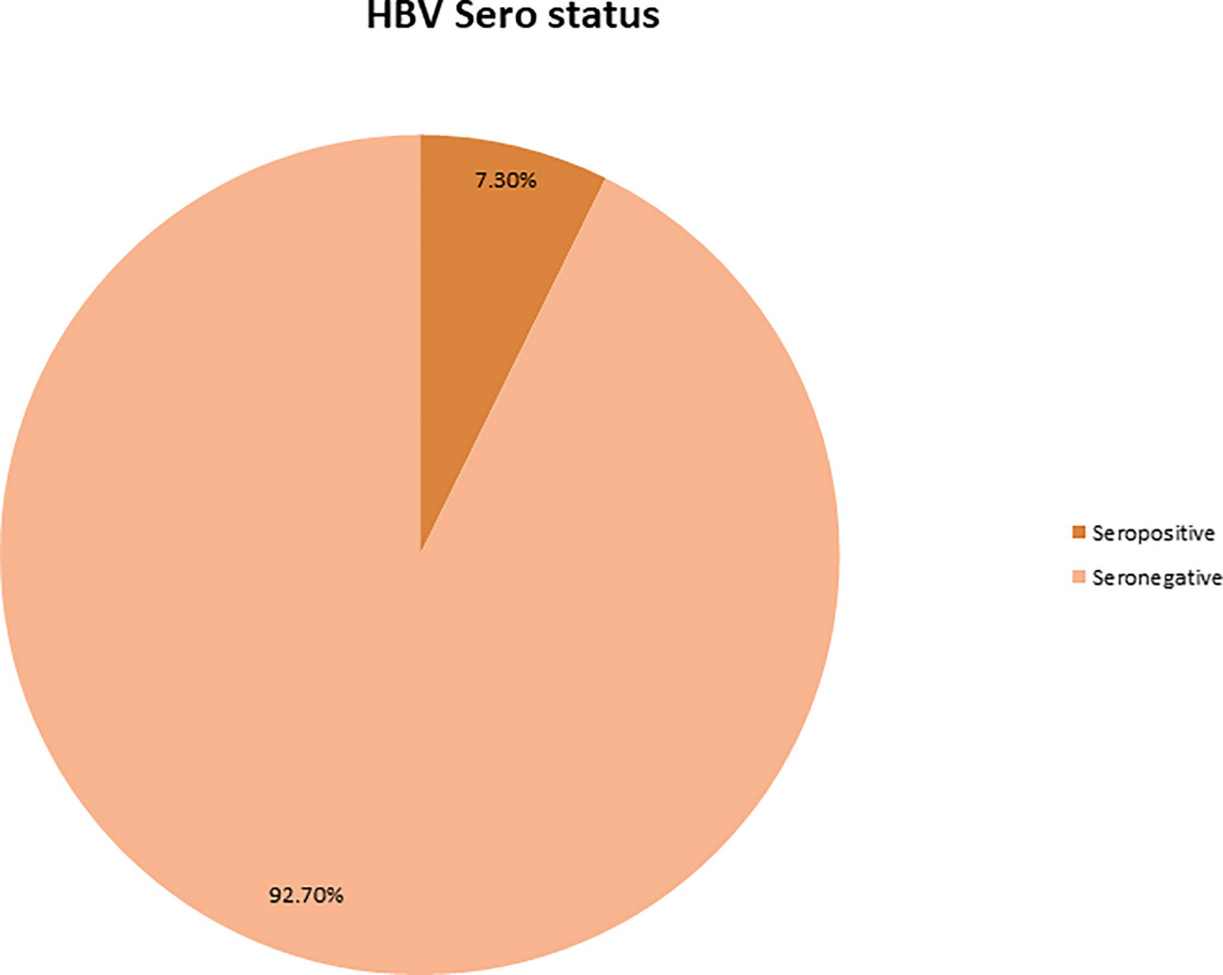
Prevalence of HBsAg among pregnant women attending antenatal clinics of public hospitals in Wolayta zone, 2018.

From total of 675 study participants, 49(7.3%) were positive for HBsAg and the rest 726(92.7%) were negative for HBsAg in laboratory test as shown in Fig 1.

### Bivariate and multivariate logistic regression result

In bivariate analysis, place of residence, history of hospital admission, history of surgical procedure, history of having multiple sexual partner, place of previous delivery, history of abortion, history of tooth extraction, history of sharing sharp materials, history of genital mutilation, family history HBV infection and body tattooing become candidate for multivariate analysis(p<0.25).

Among those entered in multivariate analysis, history of surgical procedure, history of multiple sexual partners, history of tooth extractions, and genital mutilation were significantly associated with HBV infection among pregnant women attending ANC at public hospitals in Wolayta Zone. Those participants with history of genital mutilation were nearly three times more likely to acquirep HBV infection compared to those who didn’t had mutilated (AOR = 2.7, 95%CI, (1.41–5.26)). Pregnant women having history of surgical procedure in healthy facility were 3 times more likely to acquire HBV infection comparing having no history of surgical procedure in health facility (AOR = 3.22; 95%CI (1.45–7.16)). Women who had history of multiple sexual partner were nearly three times more likely to acquire HBV infection comparing with women who had not (AOR = 2.67, 95% CI, (1.107–6.46)). Those women who had history of tooth extraction were two times more likely to acquire HBV infection comparing those women were not extracted (AOR = 2.05, 95% CI (1.06–3.96)) as shown in Table 3.

**Table 3:** Logistic regression result of variables associated with HBV Sero-status among pregnant women attending ANC at public hospitals in Wolayta Zone, 2018.

Variables (n = 675)	Category	Sero-status		COR(95%CI)	AOR(95%CI)
		Positive	Negative		
Surgical procedure	Yes	12	60	3.06[1.51–6.18]	3.22[1.45–7.16] **
	No	37	566	1	
History of multiple sexual partner	Yes	9	39	3.38[1.53–7.48]	2.67[1.11–6.46] *
	No	40	587	1	
Genital mutilation	Yes	29	463	1.96[1.08–3.56]	2.72[1.41–5.26] **
	No	20	163	1	
Body tattooing	Yes	12	72	2.5[1.24–5.01]	1.85[0.84–4.08]
	No	37	556	1	
Tooth extraction	Yes	21	157	2.24[1.24–4.06]	2.05[1.06–3.96] *
	No	28	469	1	
Abortion History	Yes	18	152	0.55[0.30–1.02]	0.57[0.62–3.27]
	No	31	474	1	
Sharing sharp material	Yes	4	21	2.56[0.84–7.78]	2.73[0.77–9.78]
	No	45	605	1	
Hospital Admission	Yes	18	179	1.45[0.79–2.66]	1.23[0.61–2.46]
	No	31	447	1	
Blood transfusion	Yes	5	102	0.43[0.27–1.51]	0.36[0.13–1.01]
	No	44	524	1	
Family history HBV	Yes	6	36	1.94[0.78–4.82]	1.53[0.56–4.14]
	No	43	590	1	

AOR = adjusted odds ratio, COR = crude odds ratio

* = <0.05

** = <0.001, CI = confidence interval

## Discussion

The overall prevalence of HBsAg in the study area was [7.3% (95%CI, (5%, 9%))]. This shows intermediate endemicity of HBV infection according to WHO classification criteria [9]. This indicates viral hepatitis infection remains a public health problem in developing countries. Factors significantly associated with HBV among pregnant women attending ANC at public hospitals in Wolayta Zone, were history of surgical procedure, history of genital mutilation, history of tooth extraction and history of multiple sexual partners.

The prevalence in this finding is higher when compared to the previous studies in different region of Ethiopia, like, 3.5% in Dawuro zone [12], 4.3% in Arbaminch Hospital [5], Dessie Referral Hospital, 4.9% [1], Adjibar Rural Health Center, Northeast Ethiopia 3.8%[15]. This might different sampling technique, sample processing techmique and existence of harmful traditional practice before years. Prevalence of current study was lower than study conducted different Africa country like, Mogadishu, Somalia 4.21%[13], upper Egypt 4.8%[16], Nairobi, Kenya 3.8%[17]. The difference could be due to difference in geographical, cultural and behavioral factors of study participants.

Current finding is comparable with study conducted in Deder Hospital, Eastern Ethiopia which was 6.8% [3], in Jigjiga which was 6% [18], in Bahar dar city, North Ethiopia which was 6.6%[19], Hawassa teaching and referral hospitals which was 7.8% [14].

Similar study conducted in different Africa countries show comparable result with current study such as Abuja Teaching Referral hospitals Nigeria 7% [20], Minna, Niger state 6.5%[21], Cross-River State, Nigeria 6.6%[7], Ijebu-Ode, Nigeria 6.7%[22], 7.5% in Khartoum teaching and referral hospital[23].

However, current finding is lower than studies done in Brazzavill, Congo which was 8.7%[24], North Eastern Nigeria where prevalence was 8.2%[7], Kogi state Nigeria which was14%[25], Juba teaching and referral hospital Republic of Sudan, where prevalence was 11% [22], the difference could be due to behavioral and cultural composition of participants.

Regarding factors, the statistically significant association was detected between HBsAg and tooth extraction of HBV (AOR = 2.05, with 95%CI = 1.06–3.96, P = 0.003) in this study. This might be due to tooth extraction practice by unsterilized instruments and sharing tooth extraction instrument without sterilization for different people in previous years in the rural area. Traditional removal of tooth by using traditional material may transfer HBV from one person to another person. This practice is conducted in the community under unhygienic conditions and sharing of equipment is common, this finding was supported by study conducted in Nigeria [26]. The current finding contradicts to the study conducted in Bahir dar city [19], Deder Hospital, Eastern Ethiopia [3], Jigjiga Eastern Ethiopia [18] on the same population. The reason could be early preventive education given to the community by health providers and mass media in the areas where tooth extraction was done under sterile condition.

In this study, participants with history of hospitalization after surgery were three times more likely to acquire HBV infection, [AOR = 3.22, 95% CI = 1.45–7.16, p = 0.004]. This finding goes in line with study conducted at Deder Hospital Eastern Ethiopia [3], but contradicted by study conducted at Arba Minch Hospital [5]. This is an indication of variation in following strict sterile procedure among hospitals that needs intervention.

History of multiple sexual partners showed statistically significant association with HBV infection. In the current study, pregnant women who had history of multiple sexual partners were almost three times more likely to acquire HBV infection as compared to their counterparts. This finding goes in line with study conducted in different parts of Ethiopia, like Yirgalem hospital [27], Deder Hospital, Eastern Ethiopia[3], Dawuro zone Southern Ethiopia[12], Arbaminch Hospital, South Ethiopia[5]. This finding is supported by science and shows risk of acquiring HBV increased by unprotected and multiple sexual partner

In this study, female genital mutilation showed statistically significant association with HBV, however, study conducted at Hawassa teaching and referral hospital and Jigjiga were contradicts current study [18, 14]. The reason could be difference in sample size and these two studies conducted in one hospital and current study were include three hospitals with large sample size compared with two studies.

## Conclusion

Intermediate endemicity of prevalence of hepatitis HBV was detected among pregnant women attending ANC in Wolaita Zone at public hospitals according to WHO classification. History of tooth extraction, history of surgical procedure, history of genital mutilation and history multiple sexual partners were factors independently associated with HBV infection in study area.

## Recommendation

Based on the study finding, we forward the following recommendations to concerned bodies in order to reduce transmission of HBV from the mother to child and its consequences.

- We recommend that health education programs on the mode of HBV transmission, especially on prevention of traditional tooth extraction, female genital mutilation, reduction of high-risk behaviors including having multiple sexual partnership and methods of preventions should be given to communities as well as to ANC attendants at antenatal care clinics to raise the awareness of mothers and community.
- It is also advisable to implement sterile procedures in every surgical procedure to prevent HBV transmission by public hospitals of Wolayta Zone.
- Legal action should be taken on those who engage in harmful traditional practice
- Further study should be performed by using more sophisticated diagnostic methods like ELISA, molecular HBV- Deoxyribonucleic acid test.

## Supporting information

S1 File (DOCX)

## Abbreviations and acronyms

ANC: Antenatal care
AOR: Adjusted Odds Ratio
CI: Confidence Interval
ELISA: Enzyme linked immune sorbent assay
HBsAb: Hepatitis B surface antibody
HBsAg: Hepatitis B surface antigen
HBV: Hepatitis B virus
WHO: World Health Organization
