# Supplementary material for: Prevalence of hepatitis B viruses and associated factors among pregnant women attending antenatal clinics in public hospitals of Wolaita Zone, South Ethiopia
Source: PLoS One. 2020 May 7;15(5):e0232653. doi: 10.1371/journal.pone.0232653 (PMC7205295; doi:10.1371/journal.pone.0232653)
Supplement: S1 File — (DOCX) [file pone.0232653.s001.docx]

Sample processing method

5 ml of venous blood was drawn under aseptic conditions in disposable vacuntainer tubes by experienced laboratory personnel. These tubes were labeled and processed at the time of collection. The blood samples taken from the participants was centrifuged at 3000 revolution per minute (RPM) for at least 10 minutes at room temperature. The rapid test was performed to deliver the result of the pregnant women at the time of screening.

**Principles:**

**Bioline HBsAg One Test:** The bioline HBsAg one test is a qualitative, solid phase, two-site sandwich immunoassay for the detection of HBsAg in serum or plasma. The membrane is precoated with anti-HBsAg antibodies on the test band region and anti-mouse antibodies on the control band region. During testing, the serum sample reacts with the dye conjugate (mouse antiHBsAg antibody colloidal gold conjugate) that will be coated in the test strip. The mixture then by capillary action reacts with anti-HBsAg antibodies on the membrane and generates a red band. Presence of this red band indicates a positive result while its absence indicates a negative result. Regardless of the presence of HBsAg, as the mixture continues to migrate across the membrane to the immobilized goat anti-mouse region a red band at the control band region will always appear. The presence of this red band serves as verification for sufficient sample volume and proper flow and as a control for the reagents

**Procedure**

1. The Bioline HBsAg test strip will be removed from foil pouch.
2. The test strip in the serum samples will be immersed with printed sample pointing toward the serum or plasma.

3. Then waiting for the red bands to appear. The test will read after approximately 15 minutes. Results after 30 minutes will not be interpreted.
 **Interpretation of the test**
**Positive-**Two distinct red bands appear, one in test region and another in the control region.
**Negative** - A single red band appears in the control region. No apparent red or pink band appears in the test region.
**Invalid** - Control band fails to appear which means improper testing procedure or deterioration of reagents probably so that the test should be repeated.
